# TA-BI: Reference Implementation in OMNeT++/INET for Reactive Routing Support in Wireless and Mobile Wireless Sensor Networks

**DOI:** 10.3390/s26206504

**Published:** 2026-10-14

**Authors:** Wiesław Jabłoński, Ksawery Krenc, Adam Kawalec

**Affiliations:** 1 WB Electronics S.A., 129/133 Poznańska Street, 05-850 Ożarów Mazowiecki, Poland; 2 Institute of Missile Technology and Mechatronics, Faculty of Mechatronics, Armament and Aerospace, Military University of Technology, 2 gen. Sylwestra Kaliskiego Street, 00-908 Warsaw, Poland; ksawery.krenc@wat.edu.pl; 3 Faculty of Mechatronics, Armament and Aerospace, Military University of Technology, 2 gen. Sylwestra Kaliskiego Street, 00-908 Warsaw, Poland; adam.kawalec@wat.edu.pl

**Keywords:** wireless sensor networks, mobile wireless sensor networks, trust-aware routing, reference implementation, OMNeT++, INET, AODV, model-to-code traceability, reproducibility, evidence provenance

## Abstract

**Highlights:**

**What are the main findings?**

- Typed state and evidence records implement the declared node, candidate and native-routing boundaries.
- Author-run deterministic probes provide a conformance record; the retained final decision fixture operates during initialization at simulation time zero.

**What are the implications of the main findings?**

- The record identifies explicit acceptance conditions and failure paths for the declared implementation profile.
- The code-free supplement supports inspection of retained evidence. Independent reproduction, network-level performance and adversarial robustness have not been established.

**Abstract:**

This article reports an OMNeT++ 6.3.0/INET 4.5.4 implementation of the TA-BI (trust-aware behavioral intelligence) observation-to-decision contract. Typed records separate current node Behavioral Information (BI), reported NeighborBI, historical node Trust and candidate-specific RouteBI and RouteScore. Evidence eligibility retains availability, age, support, confidence, provenance and version information; missing evidence is not replaced by a neutral score. The Ad hoc On-Demand Distance Vector (AODV) adapter applies native admission, destination-sequence freshness and minimum-hop precedence before TA-BI ordering, then revalidates a provisional selection or invokes native fallback. The authors’ conformance catalogue contains 104 obligations. The retained final initialization probe records two candidate admissions, one open gate, one valid provisional-winner revalidation and one final commit, and terminates at simulation time zero. These observations exercise a deterministic decision and accounting fixture, not packet forwarding in a wireless network. The code-free supplement preserves the authors’ verification record and integrity manifests but does not permit independent source rebuilding. The study supports test-based conformance for the declared profile. Independent implementation reproduction, network-level performance and adversarial robustness remain unvalidated.

## 1. Introduction

Wireless sensor networks (WSNs) and mobile wireless sensor networks (MWSNs) rely on constrained nodes to sense and relay measurements. Mobility changes neighborhoods, route lifetime and observation age. TA-BI (trust-aware behavioral intelligence) makes the eligibility of behavioral evidence explicit when supporting reactive routing [1,2,3].

Behavioral Information (BI) assesses current local packet-loss and delay quality for a next hop. NeighborBI aggregates admissible BI reports about that node, retaining reporter identity, prior Trust and age. Trust is a historical node-level assessment based on supported forwarding, link-stability and reputation evidence. These normalized assessments have distinct meanings and update horizons; they are not interchangeable probabilities.

RouteBI combines local BI, NeighborBI and route-instance stability for a next-hop–destination candidate. The Route Decision Engine combines RouteBI with node Trust to produce RouteScore. R-MISSING keeps missing, stale or otherwise ineligible evidence inactive. Ad hoc On-Demand Distance Vector (AODV) admission, sequence freshness and minimum-hop precedence apply first. RouteScore may re-rank only the resulting native tie class with a complete common evidence basis; a closed gate preserves baseline ordering, and native revalidation precedes commitment [1].

This article reports the executable mapping in OMNeT++ 6.3.0/INET 4.5.4 [4,5,6]. The novelty is the combination of typed state ownership, versioned candidates, deterministic publication and a bounded native-routing advice interface, supported by the authors’ fixed-profile conformance record.

### 1.1. Relationship to the Published Formal Contract

Article 1 defines the normative TA-BI semantics [1]; this article maps them to modules, records, interfaces and execution controls. The five-study program distinguishes formal specification (Article 1), reference implementation and deterministic conformance (Article 2), planned native functional validation (Article 3), planned quantitative comparison with baseline AODV (Article 4), and prospective original cross-study analysis of parameter dependencies, applicability and limitations (Article 5). Each study must substantiate its own claims; future work supplies no evidence here.

The direction of authority is

(1) $$\begin{array}{ccc} \mathrm{published}\;\mathrm{TA-BI}\;\mathrm{formal}\;\mathrm{specification} & ⟶ & \mathrm{executable}\;\mathrm{state}/\mathrm{module}\;\mathrm{mapping}\\ & ⟶ & \mathrm{reference}\;\mathrm{artifact}. \end{array}$$

Implementation choices cannot redefine state meanings, evidence availability, identity, native precedence or fallback. The equations in Section 3, Section 4, Section 5, Section 6, Section 7, Section 8, Section 9 and Section 10 restate executable contracts or traceability relations; Section 10.2 links them to Article 1.

### 1.2. Research Question and Analytical Objectives

**Research question.** How can the formal contract be implemented in OMNeT++/INET while preserving native AODV admission and precedence and making decision-relevant state, updates and artifact dependencies auditable?

The objectives are explicit typed ownership, coherent versioned publication, deterministic advice and fallback, and traceability from the specification to the executable profile and its retained tests.

### 1.3. Contributions

The contribution comprises (i) typed evidence and versioned node/candidate state; (ii) a native-first advice interface with common-basis gating and final revalidation; and (iii) a canonical profile tied to 104 implementation obligations and an integrity record. The recorded COMPLETE dispositions belong to the authors’ declared tests; they are not independent certification.

### 1.4. Scope and Inferential Boundary

The target is reactive multi-hop WSN/MWSN routing toward a sink, gateway or collection endpoint. General MANET deployment is not claimed, although relevant monitoring mechanisms originate in that literature [7,8,9]. The evidence concerns ownership, interfaces, formulas, configuration, build/linkage, controlled assertions and artifact identity in the declared profile. The 104 obligations are not stochastic performance samples.

Delivery, delay, throughput, energy, overhead, resource costs, scalability, mobility tolerance and adversarial robustness remain unevaluated. Native functional validation belongs to planned Article 3; quantitative benchmarking belongs to planned Article 4. Paired runs must match topology, traffic, mobility, radio/MAC settings and seeds, vary size and load, and report repeated results with uncertainty. Future studies do not extend the present claims.

### 1.5. Identifiers Used in the Evidence Record


A2_REFERENCE
Fixed canonical configuration, not a radio experiment.
A2C01–A2C13
Author-controlled conformance stages; full lineage is retained in Supplementary File S1.
R02
Final internal implementation revision.
R06
Submitted/current manuscript revisions; frozen Supplementary File S1 and R06 crosswalks are unchanged.
A2-REFSPEC-*
Requirement-catalogue revisions joining 104 stable identifiers to evidence.

## 2. Related Work and Implementation Gap

### 2.1. Trust-Aware Routing and Behavioral Evidence

Trust-aware routing research has established several mechanisms that are relevant to the executable realization of TA-BI, but these mechanisms address different semantic layers. TARF combines trust and energy information to support dependable routing in WSNs, while LDTS focuses on lightweight trust management for clustered sensor networks [2,3]. Earlier MANET mechanisms such as Watchdog/Pathrater, CORE, and CONFIDANT demonstrated the practical value of monitoring forwarding behavior, reputation, and cooperation when selecting or avoiding relays [7,8,9]. Protocol-specific trust-aware AODV variants subsequently illustrated several ways of injecting trust into reactive route selection [10,11,12,13]. These approaches motivate behavioral evidence acquisition and decision support, but they do not by themselves provide the typed observation-to-decision contract, missing-evidence semantics, candidate versioning, and bounded protocol-adapter obligations adopted by TA-BI.

Recent sensor-network routing broadens the evidence and optimization space. Trust has been combined with void avoidance, learned route selection, clustering, and multipath fuzzy logic [14,15,16,17]. Recent MANET studies likewise combine trust with localization or fuzzy inference [18,19]; they are cited here as architectural contrasts rather than as WSN-specific performance evidence. MWSN literature additionally emphasizes the effects of mobility on neighborhood continuity, route maintenance, collection strategies, and routing roles [20,21]. These developments motivate a software architecture that keeps observation meaning, temporal validity, candidate identity, and protocol integration explicit instead of embedding them in one undifferentiated routing score.

The published TA-BI formal article reviewed this lineage and defined the resulting semantic separation between current BI, reported NeighborBI, historical Trust, candidate RouteBI, and decision RouteScore [1]. The present study therefore does not repeat a broad trust-routing survey. Its implementation-specific question is narrower: how these already defined semantics can be preserved when translated into software modules, records, events, interfaces, and an AODV decision point.

Capacity and spectrum adaptation addresses another aspect of sensor-network routing. Faheem and Gungor combine capacity-aware channel assignment with fish bone routing for smart-grid WSNs [22]. Their work optimizes communication under variable link and spectrum conditions. TA-BI instead specifies the eligibility and state ownership of behavioral evidence and its bounded influence on a native routing decision. This is an architectural comparison; the smart-grid mechanism is not classified here as a trust-aware AODV variant, and its results do not establish TA-BI performance.

### 2.2. AODV Semantics and Formal Preservation

AODV uses destination sequence numbers, hop count, route lifetime, and reactive route discovery to maintain a loop-avoiding route state [23]. Formal analyses of distance-vector and AODV-family routing have shown that apparently small changes to ordering or update rules can alter safety-relevant protocol properties [24,25,26]. For this reason, the formal reference TA-BI adapter is specified not to treat RouteScore as a replacement routing metric. Native admissibility, sequence freshness, and minimum-hop precedence are required to be evaluated first; TA-BI may discriminate only inside the resulting native tie class and only when the declared evidence gate is open.

The preservation claim inherited by the implementation study is consequently bounded. The formal reference adapter requires preservation of native candidate-admissibility and precedence semantics at the TA-BI integration point; it does not assert whole-transition-system equivalence between an extended simulator and an abstract RFC model. The completed implementation supplies a frozen-epoch adapter, common-basis gate, commit-time native revalidation, and native fallback for that bounded claim. The evidence establishes the declared decision-interface behavior in deterministic conformance probes; it does not establish whole-transition-system equivalence, liveness, global loop freedom of the modified system, or preservation of every RFC 3561 property.

### 2.3. Simulation Frameworks and Reproducible Implementations

OMNeT++ provides a component-oriented discrete-event simulation environment, and INET supplies reusable protocol, mobility, wireless, and routing models suitable for implementation studies [4,6]. Their modularity is useful for separating observation, state estimation, decision advice, and protocol commitment. Framework use alone, however, does not make an experiment reproducible. Earlier reviews identified weak scenario documentation and limited repeatability in MANET simulation studies [27]; current simulation-methodology work likewise treats executable workflows and artifact identity as integral research outputs [28]. A reference implementation therefore requires an exact source revision, compatible toolchain, explicit configuration, deterministic seed policy, run commands, raw outputs, and integrity information that collectively identify the executable artifact.

This requirement is especially important for an implementation article because simulator version names do not uniquely identify the source tree, local modifications, compile options, configuration inheritance, or run state. The reproducibility contract defined later therefore treats these elements as scientific evidence rather than as incidental development metadata.

Published model libraries also provide implementation-reporting comparators. SimuLTE describes a modular OMNeT++ LTE/LTE-A simulator compatible with INET and reports performance studies [29]. Simu5G describes an OMNeT++/INET-compatible 5G library and validates model abstractions using specified scenarios [30]. Their open software connects model architecture to executable experiments. They are methodological comparators rather than WSN routing baselines: the present TA-BI supplement offers retained records but lacks the executable material needed for the same kind of reader reproduction.

### 2.4. Targeted Literature-Selection Method

The submitted implementation-specific literature update was completed on 12 September 2026 using DOI metadata and publisher platforms, including the ACM Digital Library, IEEE Xplore, MDPI, Sage, Springer Nature, and Wiley Online Library. Search combinations joined trust-aware routing, WSN, MWSN, MANET, AODV, simulation reproducibility, reference implementation, and formal verification. Records were included when they informed at least one of three boundaries: typed trust evidence, preservation of native routing semantics, or reproducibility of executable simulation studies. Pure application-performance papers were retained only when they supplied relevant architectural context. MANET results were not treated as direct evidence for WSN or MWSN behavior. This was a targeted update, not a systematic review or meta-analysis. The review-directed additions were checked against publisher or author-institution records on 7 October 2026.

### 2.5. Implementation Gap Addressed

The implementation gap addressed in this article has four parts. First, formal evidence states must become typed executable records without converting unavailable evidence into ordinary numeric values. Second, node-level and candidate-level states must retain their distinct owners, indices, horizons, and versions. Third, the Route Decision Engine–AODV boundary must preserve native protocol precedence while exposing the common-basis TA-BI comparison in an auditable way. Fourth, the internally controlled source must be tied to a reproducible build and execution record through explicit traceability and integrity controls. These four elements define the contribution of the present implementation study; quantitative superiority over baseline AODV is not part of the claim.

## 3. Reference Environment and Protocol Context

### 3.1. OMNeT++ and INET Reference Environment

The reference profile targets OMNeT++ 6.3.0 and INET 4.5.4. OMNeT++ 6.3.0 provides the discrete-event simulation kernel, module hierarchy, signals, parameters, configuration system, and runtime environments used by the implementation [4,31]. INET 4.5.4 provides the IPv4 networking stack, routing table, AODV implementation, wireless and mobility models, lifecycle infrastructure, and signal definitions consumed by TA-BI [5,6]. The selected versions are compatible according to the INET release documentation [32].

Version locking is part of the scientific artifact rather than an incidental development choice. A future result produced with a different OMNeT++ version, INET version, compiler, source revision, configuration, or seed is a new execution and must be identified as such. The final reference artifact is therefore required to record its complete environment and must not infer reproducibility from nominal software names alone.

The architecture can be mapped to other simulator releases, but binary or source compatibility is not implied. Porting requires inspection of packet APIs, signal payloads, initialization stages, routing-table interfaces, lifecycle handling and equal-time scheduling, followed by a clean build and the same positive and rejection checks. Each port requires a distinct source/environment identity. Only the recorded OMNeT++ 6.3.0/INET 4.5.4 pairing is covered by the current verification record.

### 3.2. Native AODV Responsibilities

AODV is a reactive routing protocol in which route discovery uses RREQ/RREP control messages and destination sequence numbers to represent route freshness [23]. The formal TA-BI adapter retains native authority over candidate validity, destination-sequence freshness, hop-count precedence, route lifetime, next-hop reachability, loop-prevention conditions, and route discovery or repair. TA-BI is not permitted to create feasibility where AODV has none, to retain a candidate that native revalidation rejects, or to compensate for a worse native hop count with a higher RouteScore.

This boundary is stronger than the statement that “AODV remains present” in the software stack. Conformance requires that the ordering and commitment path make native criteria observably higher priority than TA-BI advice.

### 3.3. Formal-to-Executable Mapping Problem

The implementation problem can be expressed as a traceability relation. Let *F* denote a formal construct published in the first article, *E* its executable representation, *M* the module that owns its update, *S* the storage record in which the state is published, and *C* its permitted consumer. A conformant mapping requires

(2) F↦(E,M,S,C)

This mapping permits no semantic aliasing that changes the assessed object, time horizon, availability state, or protocol precedence. Equation (2) is enforced in the release crosswalk by linking each formal obligation to its owner, publication state, consumer, and verification evidence.

The implementation further distinguishes *diagnostic availability* from *decision availability*. A scalar may be retained for continuity or logging while remaining ineligible for route comparison because its evidence is unavailable, stale, incomplete, low-confidence, or version-inconsistent. This distinction operationalizes the published R-MISSING rule [1].

## 4. Materials and Methods: Reference Implementation Architecture

### 4.1. Design Principles

The completed reference architecture is governed by seven principles. Their executable realization and evidence are bound to the 104-row traceability and closure registers.

1. **Formal-source authority.** The published TA-BI contract determines state meaning; code structure does not redefine it.
2. **Single semantic ownership.** Every primary state has one authoritative producer and one declared publication representation; producer tags, APIs, topology checks, and conformance probes enforce the boundary.
3. **Typed evidence.** A numeric value alone is insufficient for decision use; availability, resolution, time, confidence, provenance/admissibility, semantic support, support count, and version metadata are represented separately from the retained scalar.
4. **Native-protocol authority.** The AODV adapter establishes and revalidates native feasibility before TA-BI ordering can affect a candidate decision.
5. **Common-basis comparison.** The adapter compares candidates by RouteScore only when the same required components are simultaneously usable for all candidates in the native tie set.
6. **Deterministic publication.** The lifecycle coordinator orders events by (t,p,s), freezes immutable window snapshots, and publishes version-coherent typed state atomically at the declared boundary.
7. **Artifact accountability.** The internally controlled final release binds source, build state, configuration, seed, commands, raw outputs, traceability, IDE evidence, and integrity information into one version-locked artifact.

**Figure 1:**
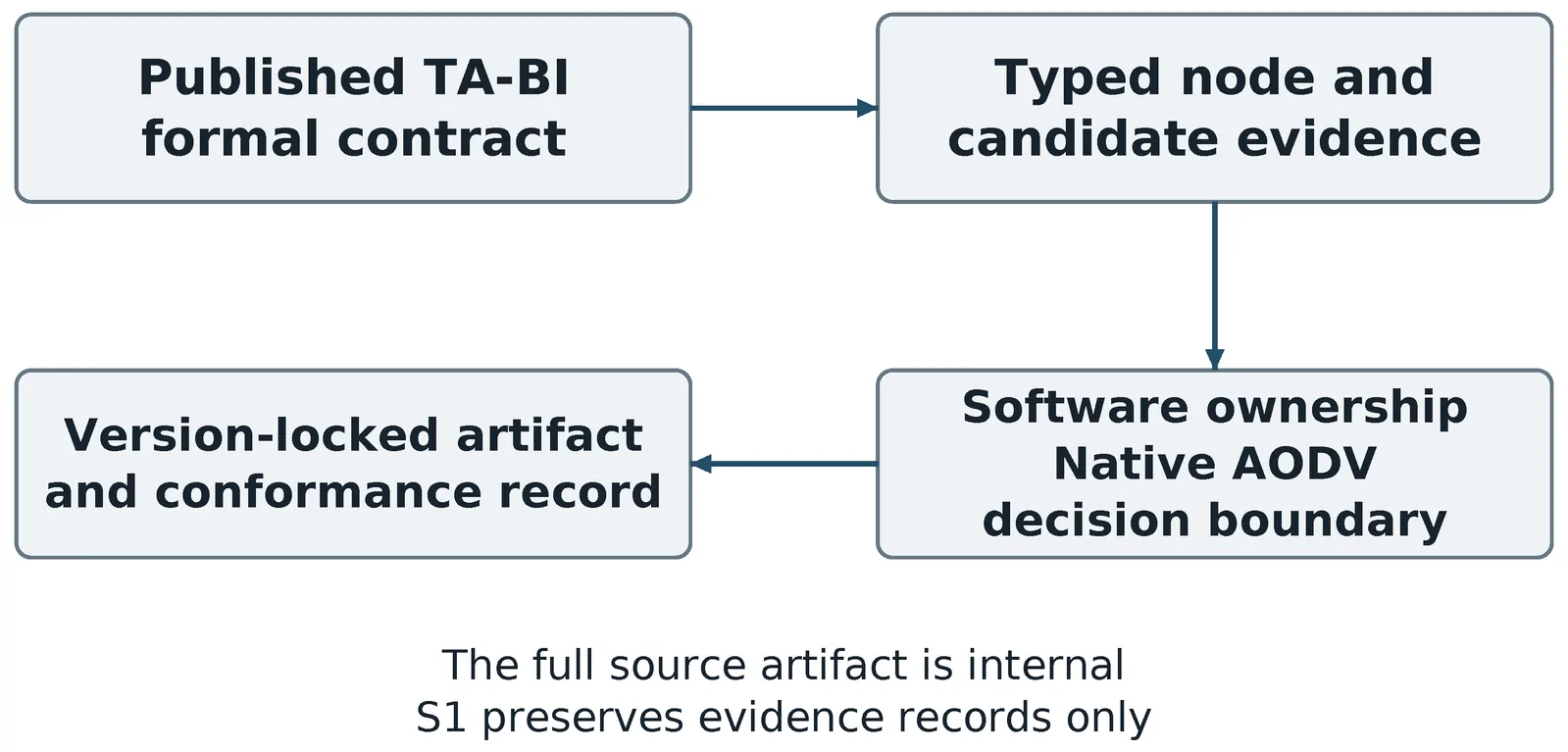
Formal-to-executable mapping under the declared test-based conformance boundary.

Figure 1 illustrates how these seven principles govern the mapping from the published TA-BI formal specification to the executable reference implementation within the declared scope of test-based conformance.

### 4.2. Module Architecture

The analytical-module count is the number of instantiated analytical NED modules in the reference composition: NodeBehavioralAnalyzer, TaBiAodvAnalyzer, DeltaManager, TrustManager, MainNodeBehavioralTable, BehavioralInformationTable and RouteDecisionEngine. Thus, the analytical count is seven. The retained final probe additionally instantiates one TaBiInstrumentationCollector and one isolated A2Conformance13Probe; neither belongs to that analytical count.

Adapter, native-port, epoch-ledger, commit-controller, lifecycle-coordinator, repository and typed-record names describe software classes or responsibilities outside the seven-module count. They must not be added to it simply because they appear in an ownership diagram. The retained initialization log and scalar typename records support the stated instance count; the full NED source topology is not distributed in Supplementary File S1.

### 4.3. Instrumentation for Subsequent Cost Accounting

The completed reference implementation exposes measurement hooks for later quantitative studies, but the existence of those hooks is not itself a performance result. The canonical instrumentation path is observational: controlled enabled/disabled runs verify that it does not alter candidate admissibility, score construction, decision timing, or route commitment. It accounts separately for native AODV and TA-BI logical control messages and bytes, all six BITReport lifecycle stages, state-table cardinalities, decision-epoch and tie-set sizes, Route Decision Engine invocations, gate outcomes, and revalidation/commit outcomes. Serialized-byte, processing-time, memory, and energy results remain outside this study.

Table 1 summarizes the verified assignment of responsibilities to modules in the completed reference implementation.

**Figure 2:**
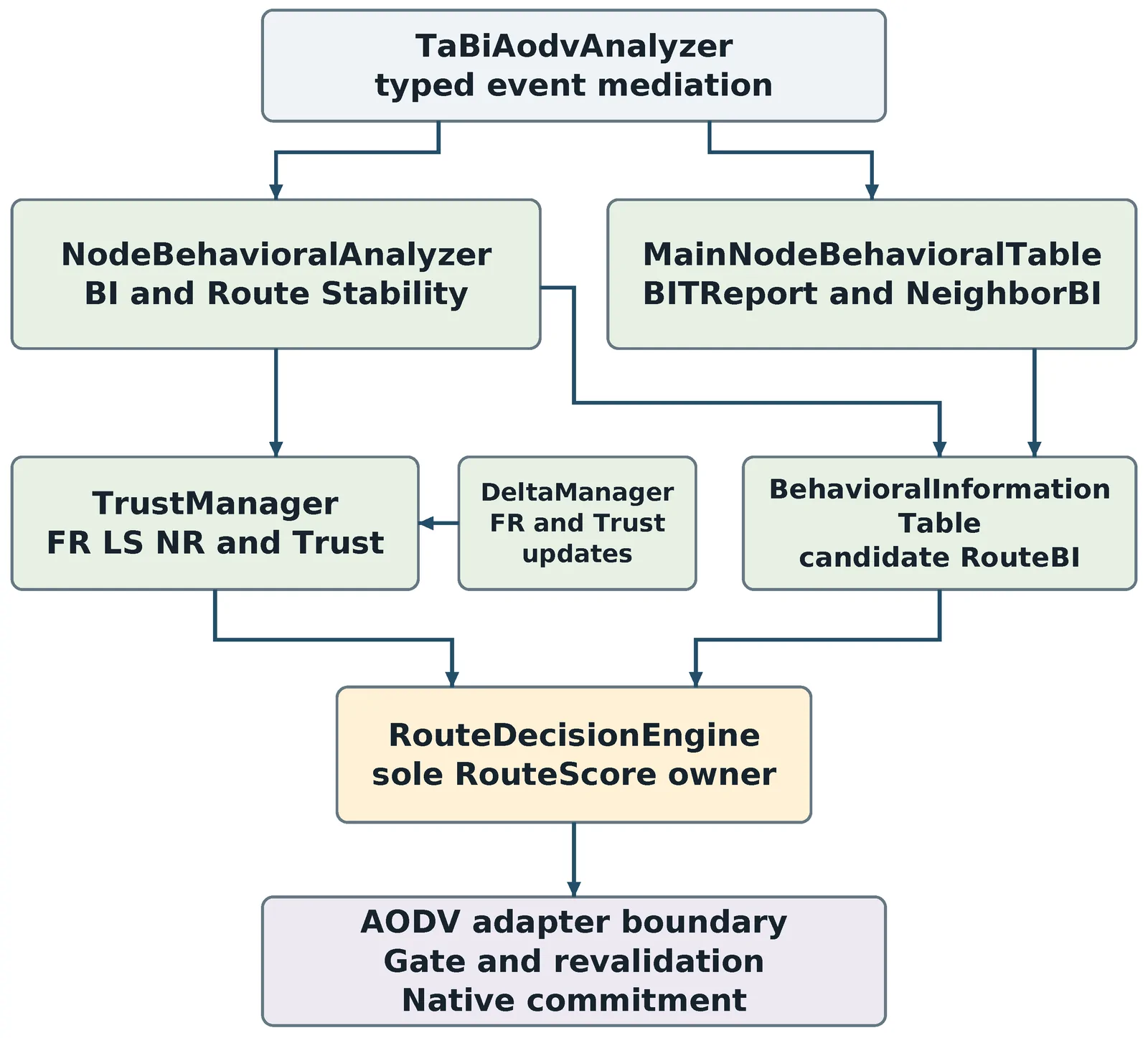
Reference software responsibilities.

Figure 2 presents the responsibilities of the seven analytical modules, the adapter boundary, and instrumentation, together with the permitted state dependencies.

### 4.4. Canonical and Experimental Profiles

The project distinguishes the canonical reference profile from experimental extensions. The internally controlled source tree defines the canonical configuration identifier A2_REFERENCE in omnetpp_A2_REFERENCE.ini. Its 66 explicit entries and production-only topology are verified by the A2C11 and A2C13 probes and by the full-release manifests.

In the complete A2_REFERENCE profile, predictive RouteScore, nonlinear score transformations, direct forwarding-ratio gates outside Trust, low-RouteScore route invalidation, validation fixtures, and other experimental adaptive mechanisms are required to be disabled unless the formal contract is explicitly extended and the extension is reported as a separate profile. This separation preserves the identity of the canonical implementation while allowing later ablation and benchmarking without deleting prior experimental research code.

## 5. Typed Evidence and State Model

### 5.1. Identity and State Levels

The executable representation preserves the assessed-object distinctions of the formal contract. Let *o* denote the local evaluator, *i* the assessed next-hop node, *j* a reporter, *d* the routing destination, c=(i,d) the candidate, and $\nu_{i,d}$ the route-instance version. The implementation represents these identities explicitly through TaBiNodeKey, TaBiReportIdentity, TaBiCandidateBaseKey, and TaBiCandidateKey. Node identity is keyed by evaluator and assessed node; candidate base identity adds destination; and a publishable candidate identity additionally requires an explicit positive route version.

The source-level state containers are TaBiNodeState and TaBiCandidateState. Node state contains BI, NeighborBI, forwarding ratio, link stability, neighbor reputation, and Trust evidence records together with a node-state version, publication serial, and publication time. Candidate state contains Route Stability, RouteBI, a Trust view, RouteScore, native route metadata, a referenced node-state version, publication serial, and publication time. The candidate Trust view records the node-state version consumed by that candidate; it does not define a destination-specific Trust update process.

### 5.2. Evidence Record

A decision-relevant scalar is represented by a typed evidence record rather than by a bare floating-point value. The minimum logical record remains

(3) $${\mathrm{EvidenceRecord}}_{X}=\left(x,a,t_{\mathrm{obs}},\Delta t,\mathrm{Conf},\pi ,\upsilon ,n\right),$$

Equation (3) separates a retained diagnostic scalar from the metadata that determines whether the scalar may participate in a decision. Here, *x* is the retained diagnostic value, a∈{0,1} denotes availability, $t_{\mathrm{obs}}$ is the last supporting observation time, Δt is age at consumption, Conf∈[0,1] is confidence, π denotes provenance/admissibility metadata, υ is compatible state or route-version information, and *n* is the supporting observation count or declared equivalent.

The logical model uses the TaBiEvidenceRecord and TaBiEvidenceMetadata types. The metadata separates available, resolved, provenanceAdmissible, and semanticallySupported from the retained scalar and records observedAt, publishedAt, confidence, provenance class, source-state version, compatible route version, semantic version, supporting-observation count, authoritative producer, producer instance, source, and semantic tag. TaBiEvidenceUsability carries the resulting usability flag, inactivity reason, and computed age. Production modules populate these fields under their declared ownership, and the controlled probes verify missing, stale, low-confidence, provenance-invalid, unsupported, and version-mismatched paths.

### 5.3. NodeState and CandidateState

The logical state separation is

(4) $$\begin{array}{cc} {\mathrm{NodeState}}_{i}^{\left(o\right)} & \;=({\mathrm{BI}}_{i},{\mathrm{NeighborBI}}_{i},{\mathrm{FR}}_{i},{\mathrm{LS}}_{i},{\mathrm{NR}}_{i},{\mathrm{Trust}}_{i},\upsilon_{i}),\\ {\mathrm{CandidateState}}_{i,d}^{\left(o,\nu \right)} & \;=({\mathrm{RouteStability}}_{i,d}^{\nu },{\mathrm{RouteBI}}_{i,d}^{\nu },{\mathrm{TrustView}}_{i}^{\nu },\\ \; & \;{\mathrm{RouteScore}}_{i,d}^{\nu },{\mathrm{NativeMeta}}_{i,d}^{\nu },\upsilon_{i,d}). \end{array}$$

The split in Equation (4) prevents a destination-specific candidate state from being mistaken for a historical node state. The TaBiNodeState and TaBiCandidateState structures implement this split, with every analytical component represented by a TaBiEvidenceRecord. TaBiNativeRouteMetadata records native admissibility, destination-sequence knowledge and value, hop count, gateway, route lifetime, decision epoch, and deterministic baseline rank. The decision adapter consumes this metadata to construct the frozen native tie class, while the commit controller revalidates native state before mutation.

### 5.4. Versioned State Repository

A dedicated TaBiTypedStateRepository provides source-level publication guards. Node-state publication requires a specified node key, a nonzero state version, and a strictly increasing version relative to any previously stored state for the same key. Candidate route versions are advanced explicitly per evaluator–assessed-node–destination base key. Candidate publication requires the repository’s current route-instance version and rejects records from earlier or otherwise non-current versions.

For candidate components marked available, the repository requires the evidence record’s compatible route version to match the current candidate version. When a candidate Trust view is available, its sourceStateVersion must match the candidate’s referencedNodeStateVersion. The decision adapter then applies the class-wide common-basis gate, and the lifecycle coordinator supplies the declared atomic event-ordering boundary.

### 5.5. Missing-Evidence Rule

The source operationalizes the published R-MISSING rule through explicit evidence usability. For component *X*, a decision-facing consumer can treat

(5) usable(X,t)=1

only when the record is available, resolved, finite, provenance-admissible, semantically supported, confidence-sufficient, sufficiently fresh, and—when required—compatible with the expected route version. Equation (5) is a gate, not a numeric imputation rule. The implemented inactivity causes distinguish missing, unresolved, non-finite, provenance-invalid, unsupported, low-confidence, stale, and route-version-mismatched evidence.

The helper activeWeightedAggregate() evaluates each candidate component through the same usability contract, omits inactive or non-positive-weight components from the active numerator and denominator, and returns no aggregate when the active-weight sum is zero. It therefore does not manufacture 0, 0.5, 1, or another neutral/favorable value when evidence is absent. The reachability probe exercises this behavior, including initialization with zero evidential confidence, stale evidence with a retained diagnostic scalar, active-weight renormalization, and an undefined empty active set.

The production BI, Trust, NeighborBI, RouteBI, and RouteScore paths use this eligibility discipline at their declared aggregation or decision boundaries; exact-oracle and degraded-evidence probes verify that missing evidence is not converted into a neutral score.

#### Illustration of Evidence Usability

Consider consumption at t=10 s with a maximum age of 2 s. An otherwise valid, resolved record with value 0.8, positive support and $t_{\mathrm{obs}}=9$ s is fresh. The same retained value supported only at $t_{\mathrm{obs}}=7$ s is stale and inactive. A retained 0.8 with no supporting observation is unavailable. This illustration is not a simulation measurement. If every weighted component is inactive, the aggregate is undefined; retaining 0.8 does not manufacture evidence or a neutral replacement.

## 6. Observation-to-State Processing

The processing rules in this section are realized by the production modules in the version-locked tabineta2 snapshot. The equations state the semantic contract, while A2C02–A2C07 provide the corresponding ownership, exact-oracle, and runtime evidence.

### 6.1. Passive Observation and Event Domains

The reference profile uses OMNeT++ signals and explicit TA-BI event interfaces to observe networking behavior without introducing a probe flow into the canonical production topology. Packet-delivery quality, delay, forwarding cooperation, local-link failure, and route-instance change remain distinct state domains and are counted separately.

The typed event contract provides evaluator, assessed node, reporter, destination, route version, causal identifier, local serial, priority, and simulation timestamp. The production observation path normalizes the subscribed INET events and uses causal identity to prevent duplicate accounting of one underlying event.

### 6.2. Behavioral Information

BI is required to represent current local communication quality of the assessed next hop. The production implementation must follow the published two-component mapping [1]:

(6) $${\mathrm{BI}}_{i}\left(t\right)=C\;\left[\frac{\sum_{q\in A_{i}^{\mathrm{BI}}\left(t\right)}\alpha_{q}Z_{q,i}^{\mathrm{BI}}\left(t\right)}{\sum_{q\in A_{i}^{\mathrm{BI}}\left(t\right)}\alpha_{q}}\right],$$

when the active-weight denominator is positive. The two canonical components are normalized packet-loss quality and normalized delay quality. Route Stability and forwarding cooperation are not BI components: Route Stability is candidate-level evidence, while forwarding cooperation contributes to Trust through FR.

BI is implemented as a current-horizon state and is not filtered by the historical update coefficient used for FR or Trust. When no admissible packet-loss or delay observation exists, the corresponding component is unavailable rather than interpreted as ideal quality. NodeBehavioralAnalyzer publishes the resulting typed BI under the canonical two-component mapping.

### 6.3. Neighbor Behavioral Information and BITReport

The complete reference profile is required to aggregate admissible reports from reporters *j* concerning assessed node *i* while preserving reporter identity and assessed-node identity as separate fields. For admissible reports, the target mapping is

(7) $${\mathrm{NeighborBI}}_{i}\left(t\right)=\frac{\sum_{j\in N_{i}\left(t\right)}\omega_{j,i}^{\mathrm{BI}}\left(t\right){\hat{\mathrm{BI}}}_{j\to i}\left(t\right)}{\sum_{j\in N_{i}\left(t\right)}\omega_{j,i}^{\mathrm{BI}}\left(t\right)},$$

when the denominator is positive, and unavailable otherwise. The reporter weight must use reporter prior Trust, report age, and provenance/admissibility checks as defined in the formal article [1].

TaBiReportIdentity and the provenance fields enforce reporter/subject separation. MainNodeBehavioralTable implements BITReport generation, transmission, reception, aging, rejection, and NeighborBI aggregation; the instrumentation ledger exposes each lifecycle stage. No communication-cost conclusion is inferred from implementation of this path.

### 6.4. Trust Assessment

Trust is required to remain a historical node-level reliability state. Direct cooperativeness is formed from Forwarding Ratio (FR) and Link Stability (LS), while Neighbor Reputation (NR) aggregates admissible direct assessments reported by other observers. The raw Trust estimate must use the active subset of FR, LS, and NR with normalized non-negative weights [1].

For forwarding ratio, a window with no expected forwarding operation supplies no forwarding evidence. Let $N_{\exp,i}>0$ denote packets successfully delivered to node *i* that should subsequently be forwarded, and let $N_{\mathrm{fwd},i}$ denote correctly resolved forwarding actions. The instantaneous observation is

(8) $${\hat{\mathrm{FR}}}_{i}=\frac{N_{\mathrm{fwd},i}}{N_{\exp,i}},$$

when $N_{\exp,i}>0$; otherwise the observation is unavailable. Stored FR and Trust must use state-specific historical updates only when the corresponding current estimate is defined; otherwise the scalar may be retained while evidence age and availability evolve independently.

TrustManager publishes FR, LS, NR, and Trust as separate typed records. DeltaManager constrains the state-specific historical coefficients, and the production path applies historical retention only to FR and Trust.

### 6.5. Route Stability and RouteBI

Route Stability is required to remain candidate-level because the same assessed next hop may participate in different route instances toward different destinations. Its production key is therefore $\left(i,d,\nu_{i,d}\right)$. Native route change, invalidation, deletion, and rediscovery events must first be normalized into candidate transitions so that one underlying transition is not counted multiple times.

When all three canonical components are available, the target RouteBI mapping is

(9) $${\mathrm{RouteBI}}_{\left(i,d\right)}\left(t\right)=C\;\left[m_{1}{\mathrm{BI}}_{i}\left(t\right)+m_{2}{\mathrm{NeighborBI}}_{i}\left(t\right)+m_{3}S_{\left(i,d\right)}\left(t\right)\right],$$

Equation (9) operates on a candidate key; Route Stability is therefore route-level rather than node-level state. The weights satisfy $m_{1},m_{2},m_{3}\geq 0$ and $m_{1}+m_{2}+m_{3}=1$. Under partial evidence, local diagnostic RouteBI may use active-weight renormalization only over defined, current, evidence-supported components. A partial diagnostic value does not by itself authorize cross-candidate comparison. NodeBehavioralAnalyzer publishes candidate Route Stability and BehavioralInformationTable computes version-compatible RouteBI; the common-basis gate remains the decision-eligibility authority.

### 6.6. State-Specific Historical Update

The canonical profile must not use one shared low-pass update over all TA-BI quantities. Historical bounded updates apply only to states that formally retain memory. In the target production mapping, FR and Trust use independently identifiable state-specific coefficients within declared bounds. BI and NeighborBI represent the current evidence horizon; Link Stability and Route Stability derive from event windows; RouteBI is the current candidate aggregation; and RouteScore remains unfiltered.

DeltaManager is restricted to admissible state-specific historical coefficients and does not propagate one global coefficient into BI, NeighborBI, RouteBI, or RouteScore.

## 7. Route Decision Engine and Typed Decision Advice

### 7.1. Single RouteScore Authority

The reference profile assigns the Route Decision Engine (RDE) sole authority for decision RouteScore. No observer, analyzer, storage table, or AODV event mediator calculates an alternative decision RouteScore. The production RouteDecisionEngine emits typed advice with ROUTE_DECISION_ENGINE authority and the A2C06–A2C07 probes verify the exact formula, evidence audit, readiness states, and absence of apply/commit authority.

For the canonical candidate (i,d), the required RouteScore mapping is

(10) $${\mathrm{RouteScore}}_{\left(i,d\right)}\left(t\right)=C\;\left[\lambda_{\mathrm{BI}}{\mathrm{RouteBI}}_{\left(i,d\right)}\left(t\right)+\lambda_{\mathrm{Tr}}{\mathrm{Trust}}_{i}\left(t\right)\right],$$

Equation (10) is evaluated only by the RDE, which prevents alternative decision scores from emerging elsewhere in the pipeline. The coefficients satisfy $\lambda_{\mathrm{BI}},\lambda_{\mathrm{Tr}}\geq 0$ and $\lambda_{\mathrm{BI}}+\lambda_{\mathrm{Tr}}=1$, and the required inputs must be available, current, confidence-sufficient, provenance-admissible, and version-compatible. The canonical production RouteScore must not receive additional predictive, nonlinear, forwarding-ratio-gate, or history-filter transformations.

### 7.2. Decision Completeness and Component Confidence

The RDE publishes, for each configured RouteScore component, the value together with availability, last supporting time, support count or declared equivalent, confidence, provenance/admissibility, source-state version, and compatible route version. Candidate completeness and decision eligibility remain distinct: a local diagnostic score may exist without being authorized for candidate comparison.

The implemented score interface distinguishes unavailable, diagnostic-only, and decision-ready conditions. Production computation and record population are verified by exact-formula, audit, readiness, stale-evidence, confidence, provenance, and version probes.

#### Diagnostic and Decision Use

A candidate may retain a partial RouteBI for diagnostics after NeighborBI becomes unavailable. That retained value does not establish complete RouteBI support. If a required RouteBI or Trust input is unavailable, the RDE produces no canonical RouteScore. If candidate A is individually ready but another member B of the same native tie class lacks a required component, the common-basis gate still closes for the whole class. Individual advice readiness therefore does not replace class-wide activation. Baseline ordering remains available subject to native revalidation.

### 7.3. Typed Route Advice

The current TaBiApi source defines a concrete typed route-advice record. TaBiRouteAdvice contains candidate identity; BI, RouteBI, Trust, and RouteScore evidence records; native route metadata; explicit advice status; valid and apply flags; and a diagnostic reason string. Its implemented status vocabulary comprises UNSET, DISABLED, INVALID_IDENTITY, EVIDENCE_UNAVAILABLE, EVIDENCE_STALE, LOW_CONFIDENCE, PROVENANCE_INVALID, VERSION_MISMATCH, DIAGNOSTIC_ONLY, and DECISION_READY.

The logical boundary remains

(11) $${\mathrm{RouteAdvice}}_{i,d}^{\nu }=\left(\mathrm{RouteBI},\mathrm{Trust},\mathrm{RouteScore},a,\mathrm{Conf},t,\pi ,\nu ,\mathrm{status},\mathrm{reason}\right).$$

The typed interface removes the need to manufacture a neutral scalar merely to populate an advice object. The production RDE is the authoritative advice producer; application and commitment remain exclusively on the native adapter side of the boundary.

## 8. Reference AODV Adapter

The AODV adapter described in this section is implemented by the decision adapter, epoch ledger, native-port boundary, and commit controller. A2C08–A2C10 verify native admission, tie-class precedence, common-basis gating, closed epochs, revalidation, atomic commitment or fallback, and deterministic lifecycle ordering.

### 8.1. Adapter Boundary and Decision Epoch

The adapter boundary is implemented by TaBiAodvDecisionAdapter, TaBiAodvEpochLedger, TaBiAodvNativePort, and TaBiAodvCommitController. The integration admits only candidates accepted by native AODV rules and revalidates native conditions immediately before commitment.

For each destination *d* and decision epoch *ℓ*, the adapter is required to define a bounded RREP collection interval

(12) $$E_{d,\ell }=\left[t_{d,\ell }^{\mathrm{open}},t_{d,\ell }^{\mathrm{close}}\right],$$

and to freeze the admitted candidate set at closure according to the declared equal-time ordering policy. The frozen set provides a deterministic comparison domain; it does not claim timing equivalence with an AODV implementation that commits an earlier RREP immediately.

### 8.2. Native Freshness and Hop-Count Tie Class

Within the frozen native-admissible set, the adapter first retains candidates with the freshest destination sequence number using the native freshness relation or a resolved equivalent supplied by INET. Among those candidates, it retains the minimum-hop subset. The resulting set is the native tie class $C_{d,\ell }^{\mathrm{tie}}$.

TA-BI RouteScore is not considered before this tie class exists. Consequently, a higher RouteScore cannot compensate for a stale destination sequence number or an additional hop. Adapter audit records and deterministic probes make this precedence inspectable.

### 8.3. Common-Basis Activation Gate

For each candidate *c* in $C_{d,\ell }^{\mathrm{tie}}$, let $A_{c,q}\in \left\{0,1\right\}$ denote whether configured RouteScore component *q* is available, fresh, confidence-sufficient, provenance-admissible, and version-compatible. The required common component mask is

(13) $$M_{d,\ell ,q}=\prod_{c\in C_{d,\ell }^{\mathrm{tie}}}A_{c,q}.$$

The canonical complete-basis gate is required to open only when every configured positive-weight component is common to every candidate in the tie set:

(14) $$\Gamma_{d,\ell }=1\;⟺\;M_{d,\ell ,q}=1\;\mathrm{for}\;\mathrm{every}\;\mathrm{configured}\;q.$$

Otherwise, $\Gamma_{d,\ell }=0$ and TA-BI re-ranking is disabled for that epoch. No candidate receives a missing-data penalty and no unavailable component is replaced by a neutral score. The implementation combines per-record usability and route-version guards with the class-wide gate.

### 8.4. Deterministic Ordering and Baseline Equivalence

When $\Gamma_{d,\ell }=1$, the adapter compares candidates inside the native tie class using RouteScore. If RouteScore is equal within the declared comparison semantics, deterministic baseline rank resolves the tie. The reference ordering is lexicographic in the following priority:

(15) $$\mathrm{destination-sequence}\;\mathrm{freshness}≻\mathrm{lower}\;\mathrm{hop}\;\mathrm{count}≻\left(\Gamma \cdot \mathrm{RouteScore}\right)≻\mathrm{baseline}\;\mathrm{rank}.$$

Equation (15) makes RouteScore subordinate to both native freshness and hop count. When the gate is closed, the third component is inactive and the ordering over the same frozen tie set must reduce to the declared baseline ordering.

**Figure 3:**
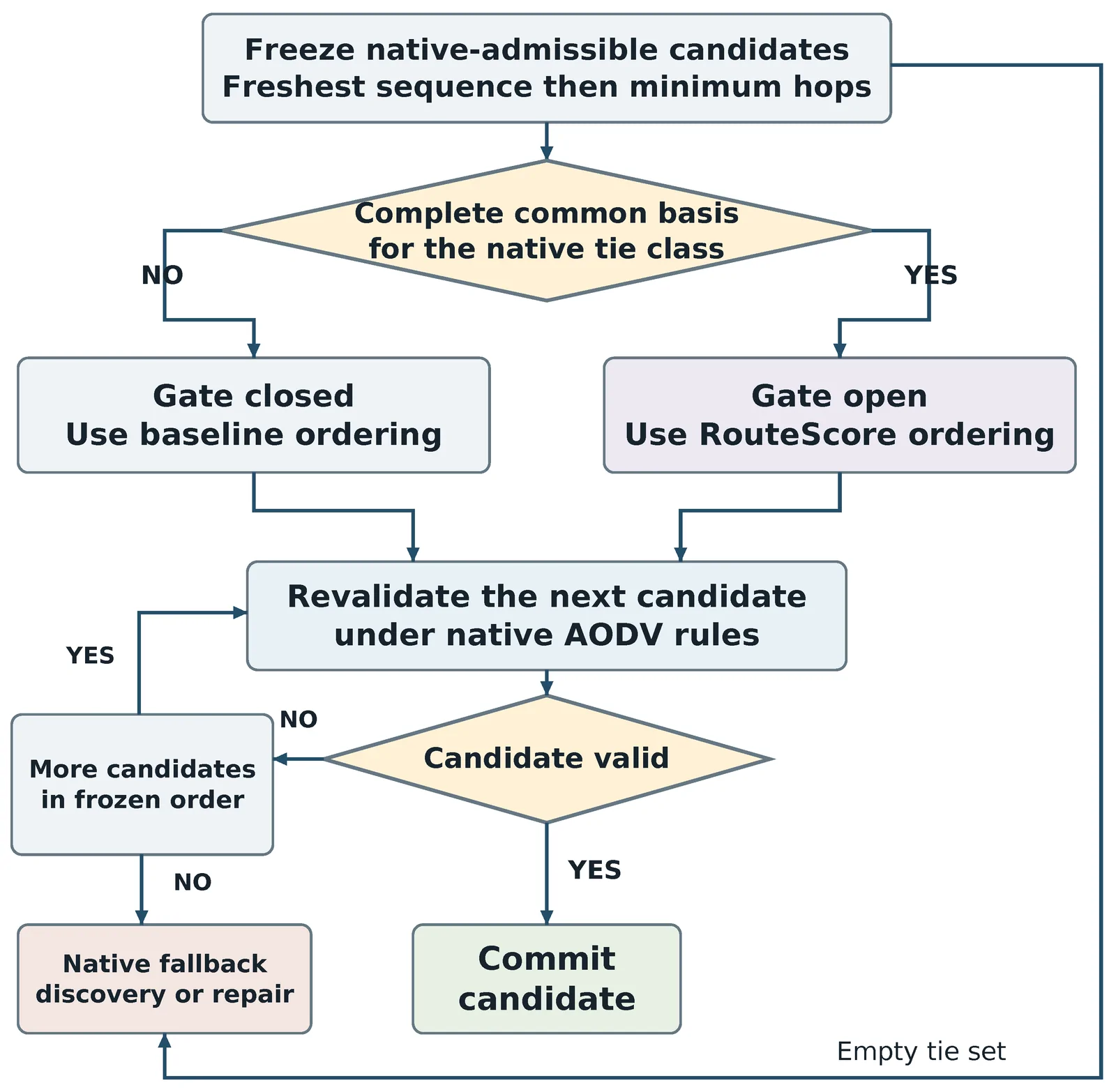
Reference adapter decision paths.

Figure 3 illustrates the adapter decision paths, including native revalidation for both gate outcomes, baseline ordering when the gate is closed, and native fallback when the candidate set is empty or exhausted.

### 8.5. Hysteresis, Dwell Time, and Late RREPs

In the complete profile, hysteresis must apply only when the incumbent and challenger remain members of the same current native tie class and the common gate is open. A TA-BI-induced switch may occur only when the challenger’s score exceeds the incumbent by the configured switching margin $\varepsilon_{\mathrm{sw}}$ and any minimum dwell time has elapsed. Fresher sequence information, a lower native hop count, route invalidation, or another higher-priority native condition must bypass TA-BI hysteresis.

A late RREP follows the declared bounded-epoch rule and cannot retroactively alter a closed decision epoch; the epoch ledger records and verifies this behavior.

### 8.6. Final Revalidation and Native Fallback

Immediately before installation or switching, the complete adapter must revalidate candidate membership, destination-sequence freshness, next-hop reachability, route lifetime, route-instance version, and all relevant native AODV conditions. If a provisional winner fails revalidation, the adapter must evaluate the next eligible candidate under the declared frozen ordering; exhaustion must invoke the native AODV discovery or repair path.

The target adapter result is total:

(16) $${\mathrm{Decision}}_{\mathrm{AODV}}=\mathrm{Select}\left(c\right)|\mathrm{Fallback}\left(r_{f}\right),$$

Equation (16) guarantees one terminal interface outcome: a revalidated candidate or an explicit native fallback reason. Here, $r_{f}$ records the native reason for fallback. A closed evidence gate is not itself a route failure; it disables TA-BI re-ranking and preserves native/baseline decision availability.

Low RouteScore alone does not mark an otherwise native-admissible route as broken in A2_REFERENCE. The canonical profile also does not translate RouteScore into a hop-count or routing-table metric penalty. Static and runtime assertions verify both exclusions.

## 9. Deterministic Execution, Versioning, and Lifecycle Semantics

### 9.1. Typed Event Identity and Equal-Time Ordering

TaBiEvents.h defines the event identity consumed by the deterministic execution policy. TaBiEventIdentity carries evaluator, assessed node, reporter, destination, route-instance version, causal identifier, local serial, semantic priority, and simulation timestamp. Packet and route event types are represented separately. These fields construct the ordering key

(17) $$\kappa \left(e\right)=\left(t_{e},p_{e},s_{e}\right),$$

Equation (17) resolves equal-time events through semantic priority and then a monotone local serial number. Here, $t_{e}$ is simulation time, $p_{e}$ semantic priority, and $s_{e}$ a monotone local serial number.

The production lifecycle coordinator enforces the equal-time priority policy across observation, route-version invalidation, evidence publication, RREP admission, epoch closure, and route commitment. A2C10 verifies the declared (t,p,s) ordering, serial monotonicity, immutable window snapshots, and one terminal outcome per epoch.

#### Equal-Time Illustration

At one local timestamp *t*, the frozen source assigns priorities 10 to route-instance invalidation, 20 to RREP admission, 60 to candidate-state publication and 70 to RREP-epoch closure. The local comparator processes lower priorities first: invalidation precedes candidate publication, and admission precedes closure. Two RREP admissions with the same *t* and priority 20 and local serials *s* and s+1 are processed in that serial order. These constants were checked in the internal TaBiLifecycleCoordinator source. The example illustrates local lifecycle ordering, not an order shared across distributed nodes.

### 9.2. Versioned Publication and Atomicity Boundary

TaBiTypedStateRepository enforces the publication guards: node-state versions increase strictly for a node key; route-instance versions advance explicitly per candidate base key; candidate records are accepted only for the current route version; and available candidate evidence is compatible with that version. Candidate Trust views retain the consumed node-state version.

These repository guards prevent an explicitly stale candidate version from entering a current state. The lifecycle coordinator adds atomic observation-window closure, same-time route-version advancement, and one version-coherent publication transaction across the declared production chain.

### 9.3. Lifecycle and Degraded-Evidence State

The complete reference profile must treat module loss, evidence timeout, missing reports, unsupported observations, and version mismatch as analytical-state degradation rather than automatic native route failure. Loss of a required evidence basis must close the relevant TA-BI decision path while preserving native route availability whenever AODV still considers a route admissible. A later complete, fresh, version-consistent basis may affect only a later eligible decision epoch.

The evidence model represents degradation through explicit inactivity reasons without discarding the retained diagnostic scalar. The production gate closes TA-BI enhancement for the affected tie set while native AODV availability remains independently governed. Restored evidence becomes eligible only in a later version-coherent decision epoch.

## 10. Model-to-Code Traceability

### 10.1. Traceability Dimensions

The implementation study treats traceability as an evidential product rather than merely a source archive. For each formal obligation, the complete traceability register is required to identify the source formal construct, manuscript section, target implementation owner, source-code symbol or location, canonical configuration dependency, artifact/source revision, and verification evidence:

(18) $$\mathrm{Trace}\left(r\right)=\left(F_{r},S_{r},M_{r},C_{r},P_{r},V_{r},T_{r}\right).$$

Equation (18) is materialized by the 104-row machine-readable claim crosswalk supplied with the article. The relation is intended to be bidirectional: from a formal construct, a reviewer should be able to find its executable representation and later acceptance evidence; from a code path that affects decision state, the authorizing formal rule should be identifiable.

The 104 obligations were derived by the authors by decomposing the published contract into executable ownership, state, evidence, observation, aggregation, decision, adapter, lifecycle, artifact and instrumentation conditions. The category sizes are architecture 7, state 6, evidence 8, observation 5, BI 6, updates 5, Trust 8, NeighborBI 7, route state 6, decision 8, AODV 15, execution 7, artifacts 10 and instrumentation 6. Their sum is 104. These are implementation obligations selected by the authors, not independent statistical samples or an externally certified standard.

The authors also defined the stimulus/oracle matrices and performed the reported acceptance checks. Acceptance is test-specific: for example, missing required input must not produce RouteScore; an inadmissible candidate must not enter selection; a larger score must not override minimum-hop precedence; a wrong route version must not replace coherent state; and failed native revalidation must prevent commitment. The corresponding decision, adapter and lifecycle matrices identify these rejection conditions. A recorded PASS or COMPLETE means that the authors’ declared checks were satisfied. It does not establish model checking, independent replication, exhaustive state-space coverage or mutation testing of all 104 requirements.

### 10.2. Equation-Level Formal Crosswalk

Table 2 binds all 18 numbered equations in this implementation article to the controlling equations, rules, or semantic sections of Article 1 [1]. The relation column distinguishes inherited semantics from implementation-only representation and traceability constructs. A machine-readable row-per-equation version is supplied as A1_A2_EQUATION_CROSSWALK_R06.csv.

### 10.3. Reference Ownership Mapping

Table 3 summarizes the verified ownership contract of the completed profile. The 104-row supplementary crosswalk provides the requirement-level source and evidence detail.

### 10.4. Complete Source and Evidence Trace

The frozen requirements catalogue contains 104 unique A2-REQ-* identifiers. The machine-readable R06 crosswalk joins each obligation to its Article 1 section, implementation work package, concrete source evidence, conformance state, runtime/IDE evidence basis, and R06 manuscript anchor. All 104 rows are MAPPED and COMPLETE; historical milestone wording is retained only as provenance and does not override the final global status.

Table 4 summarizes the traceability and release evidence for the internally controlled full A2 package.

## 11. Controlled Reference Artifact and Publication Evidence

### 11.1. Canonical Environment and Clean-Build Contract

The authoritative environment is OMNeT++ 6.3.0 build 251110-bb3d6a4731 with INET 4.5.4 on Ubuntu 24.04 under WSL2, Linux kernel 6.6.87.2-microsoft-standard-WSL2. The release Make database identifies ccache clang++. Here, ccache 4.9.1 is the wrapper at /usr/bin/ccache, while Clang 18.1.3 is the resolved compiler at /usr/lib/llvm-18/bin/clang. The evidence records the command path, resolved path, version, and binary SHA-256 for both programs. The controlled sequence regenerates the Makefile, executes mandatory make clean, builds in release mode, and verifies dynamic linkage to INET and OMNeT++ libraries. The authors also retained an IDE debug-build observation; it is not a third-party reproduction.

### 11.2. Canonical A2_REFERENCE Configuration

The canonical profile contains 66 explicit contract entries: 51 network-to-submodule bindings, five decision-boundary values, and ten exclusion guards. Table 5 reports the operational groups; the complete key/value list is distributed in A2_REFERENCE_DECISION_CONTRACT.csv and copied into the article traceability supplement.

The numerical values are reference conformance anchors. They are not asserted to be optimal and do not replace calibration, sensitivity analysis, or paired performance evaluation.

These fixed weights are chosen as reproducible formula-test settings: positive coefficients expose every configured component, while unequal Trust and RouteBI/RouteScore coefficients exercise weighting and active-set renormalization. They are not calibrated resource or performance optima. The present record therefore justifies exact conformance at those settings, not their suitability for every deployment. A later sensitivity study must vary weights on their non-negative unit-sum domains, include component ablations and report whether gate activation and native-tie decisions change across paired network runs.

### 11.3. Run Identity, Raw Outputs, and Integrity

The authoritative internally controlled bundle is TABINETA2_A2_CONFORMANCE_13_R02.zip, SHA-256 a7b8c8afcba66e8917f93e992cdf3a50bd424379b5a2cc6e8cf31c94ea666760; it is not distributed with this article. The A2C12 run identity fingerprints source, configuration, complete INET source state, compiler state, OMNeT++/OS state, seed 20260910, run number 0, and release mode. The reference configuration runs under Cmdenv and intentionally terminates at simulation time zero as a composition, configuration, and identity anchor. A2C13 separately retains its runtime log and raw .sca, .vec, and .vci files.

The full internal package passed 69/69 source hashes, 345/345 release-input hashes, 1186/1186 package hashes, ZIP CRC, path-safety, and independent re-extraction. Generated objects, libraries, executables, generated Makefiles, and installed-tree results are absent from its controlled source surface. Supplementary File S1 is a separate publication derivative, TABINETA2_A2_CONFORMANCE_13_R02.zip, SHA-256 64bcabcb6e8e310dbd1c717f8b5a610ddeddda1231540797387e2f6c88fffec7. It contains 917 non-executable evidence, traceability, runtime-output, status, and IDE-image files. It contains no TA-BI project source code, NED/MSG model source, simulation configuration, build or verification scripts, patches, IDE/build metadata, compiled artifacts, or nested archives. Supplementary File S1 therefore supports inspection of the retained verification record but not independent source rebuilding.

Independent rebuilding requires the exact C++ and header sources, NED/MSG models, configuration inheritance, build inputs, dependency pins and executable verification scripts matching the frozen release. Those materials are absent from Supplementary File S1. Source-file hashes and screenshots cannot replace them. No public source repository, third-party build, independent implementation comparison or model-checking result is claimed here. The ability to reproduce methods and outputs is distinct from having already completed a third-party replication. Supplementary File S1 enables record inspection but not source rebuilding, and this source-access limitation remains substantive even when every retained evidence-file hash verifies.

**Figure 4:**
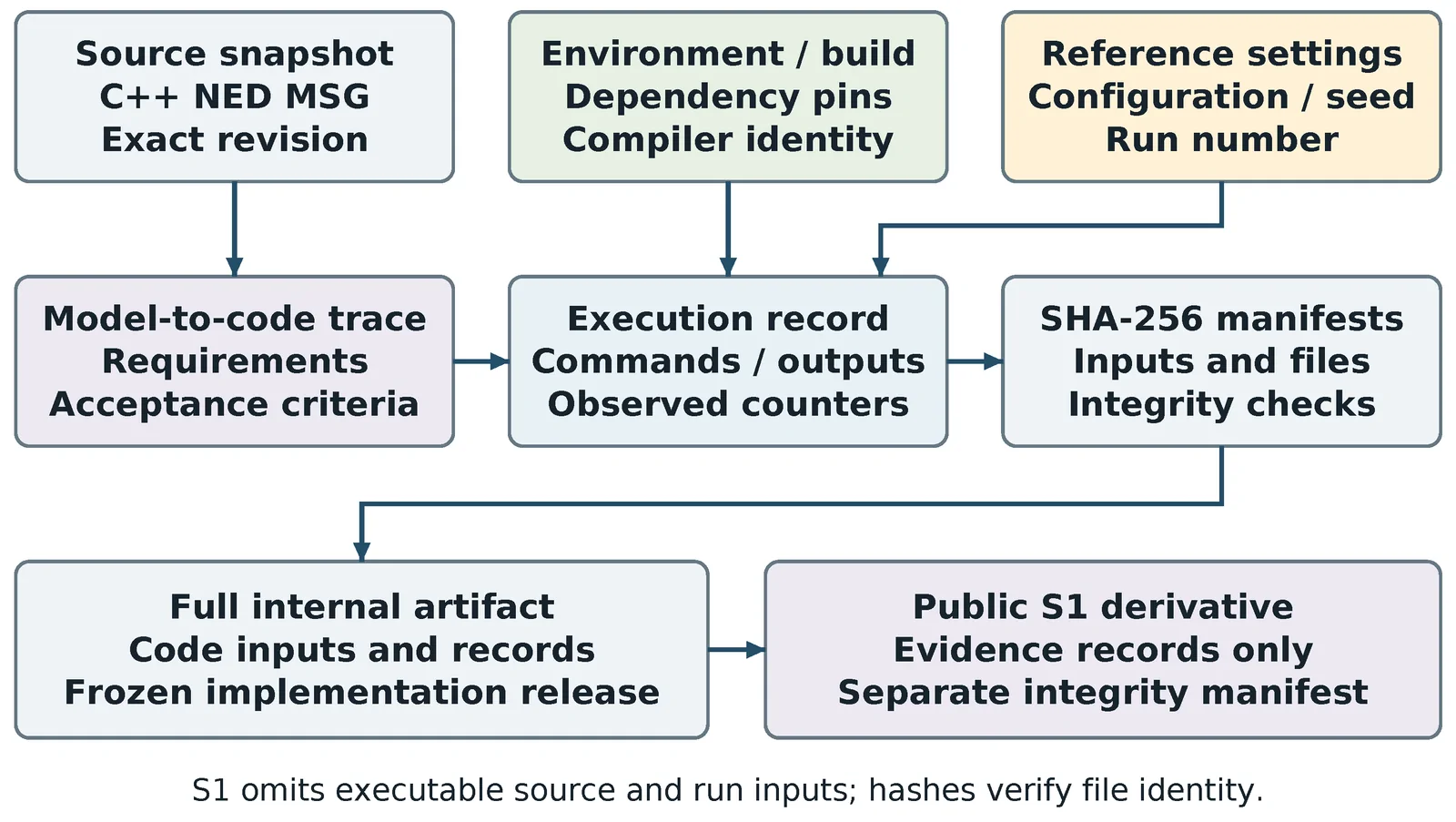
Full internal reference artifact.

Figure 4 illustrates the relationship between the full internal reference artifact and its public derivative, Supplementary File S1, which contains no source code.

## 12. Verification Results and Study Boundary

### 12.1. Verified Implementation-Conformance Evidence

The authors’ A2C01–A2C13 register records all 104 implementation obligations as COMPLETE under its declared acceptance checks. Its final terminal summary reports zero return codes for static checks, Makefile generation, cleaning, build, clean-build assertion, linkage, parent regression, A2_REFERENCE reachability, A2C13 runtime, exact pass assertion, and raw-output retention. The final probe confirms separately observable native AODV and TA-BI control accounting; all six BITReport lifecycle stages; state and report cardinalities; decision, epoch, tie-set, gate, and revalidation counters; and equality of decision and commit outcomes with instrumentation enabled and disabled.

IDE screenshots establish that the tabineta2 project references inet4.5, does not reference legacy tabinet, and builds the debug shared library with zero errors and zero warnings. These author-recorded observations complement the clean release build and runtime record; they do not constitute third-party verification.

### 12.2. Retained Final Probe and Numeric Observations

The retained final run is A2_CONFORMANCE_13, run number 0, repetition 0, seed set 20260910, under Cmdenv. Its configuration inherits the canonical profile and sets a simulation-time limit of 0 s. The log places the probe verdict in initialization, followed by the end-simulation event at t=0. It records a composition of seven analytical modules, the collector and the isolated probe. This is a module graph; it does not encode a physical-node count, radio topology, application traffic or mobility experiment.

The accounting test uses declared lengths for one RREQ, one RREP, one RERR and one TA-BI message. The six BITReport counters are exercised as distinct accounting operations; they do not prove an over-the-air report lifecycle. Closed-gate and failed-revalidation rejection conditions occur in the separate adapter test matrices, not as nonzero outcomes in this final probe. These numeric observations make the retained conformance record inspectable but do not supply a positive-duration wireless-network experiment or third-party reproduction.

Table 6 reports the exact retained observations from the final initialization probe; candidate and message counts refer to controlled calls rather than native-radio packet measurements.

### 12.3. Boundary to Functional Validation

The present deterministic probes test the release at the declared implementation boundary. The planned third study will address scenario-level functional validation in native wireless-network simulations. Its scope includes naturally produced BI and Trust, BITReport generation and reception through the radio stack, and isolation of native buffers and active decision epochs. Mobility-induced candidate turnover, degraded forwarding, candidate loss, evidence restoration and repeated route lifecycle sequences will be evaluated under explicitly identified configurations to establish functional outcomes and model conformity. Statistical comparison of performance, scalability, resource costs and resilience under network stress is reserved for the planned fourth study. These are planned investigations, not results of the present study. Agreement with specified deterministic test outcomes does not establish complete native-network operation, and future experiments cannot retroactively validate the present conformance record.

### 12.4. Boundary to Quantitative Benchmarking

Quantitative comparison with baseline AODV requires paired repeated experiments with identical topologies, traffic, mobility traces, attack schedules, seeds, radio/MAC settings, and build state. Packet delivery, delay, route dynamics, energy, serialized communication overhead, processing time, memory, scalability, and sensitivity therefore remain outside the present result. Instrumentation provides the counters needed for later accounting but does not itself establish that any cost is low.

## 13. Discussion

### 13.1. Implementation Contribution

The reported implementation addresses the formal-to-executable mapping at the declared reference-profile boundary. Its design constrains semantic drift by separating current and historical state, node and candidate identity, scalar retention and evidence eligibility, calculation and storage ownership, analytical advice and native routing authority, and conformance instrumentation and decision state. These separations are enforced by typed records, producer tags, version guards, constrained APIs, isolated production topology, deterministic probes, and machine-readable traceability.

### 13.2. Canonical Profile and Experimental Extensions

The canonical profile explicitly excludes neutral fallback, periodic advice refresh, forwarding-ratio gates outside Trust, score-driven switching transformations, nonlinear RouteScore mapping, consistency aggregation, predictive correction, controlled drop, synthetic traffic, and validation fixtures. This preserves TA-BI-T/TUNE work as separately named experimental profiles without allowing it to redefine the Article 1 contract.

### 13.3. Candidate-Admissibility Preservation Boundary

The implemented adapter supports the bounded property requested in the prior review: candidates originate inside the declared native-admissible frozen set; destination-sequence freshness and minimum-hop precedence are applied before RouteScore; a closed gate returns native baseline ordering; and the provisional winner is revalidated before one atomic commit or native discovery/repair fallback. This is candidate-admissibility preservation and native fallback, not timing equivalence, event-trace equivalence, liveness, global loop freedom of the modified system, or preservation of every RFC 3561 property.

### 13.4. Reproducibility and Evidence Separation

The full internal package binds the authors’ execution record to a controlled artifact identity. Its source, configuration, dependency/compiler identity, commands, seed/run identity, raw outputs, terminal/IDE evidence, deltas, and manifests are bound together. Public Supplementary File S1 preserves the non-executable evidence subset and its own manifest while withholding the project source and executable workflow. This strengthens the audit trail for the implementation claims without representing Supplementary File S1 as an independently rebuildable artifact or converting conformance evidence into a performance benchmark.

### 13.5. State Growth and Resource Accounting

Resource use depends on the number of retained assessed nodes, candidate versions, observation windows, pending forwarding identities and reports. The configured candidate cap bounds one comparison set; it is not a demonstrated bound on total resident memory. Measuring memory requires record sizes, container and allocator overhead, retention rules and peak resident state. Processing cost must be measured with documented instrumentation overhead and repeated workload conditions. No low-memory or low-processing-cost conclusion follows from a table-cardinality sample.

Radio overhead must count serialized TA-BI payloads together with transport, network and MAC headers, retransmissions and transmission frequency, separately from native AODV control traffic. The final ledger’s declared 80 and 144 byte inputs only check category separation. They cannot estimate airtime, contention, energy or comparative on-air overhead. Scaling studies must vary network size and load while reporting retained-state growth, execution time, gate frequency and radio costs.

### 13.6. Adversarial Reporting and Security Scope

Controlled rejection of malformed provenance, duplicated events, stale reports and incompatible versions is within the deterministic conformance scope. Empirical resistance to adversarial evidence injection, replay, collusion, Sybil behavior and reporter compromise is outside the evaluated scope. Simulator reporter identity and provenance metadata are assumptions at the model boundary; they do not establish cryptographic authentication. A compromised or colluding reporter may submit a plausible false report with otherwise valid metadata. Reporter-Trust weighting is a semantic aggregation rule, not a demonstrated defense against these attacks.

### 13.7. Threats to Validity

The verified profile is specific to OMNeT++ 6.3.0, INET 4.5.4, the recorded compiler and environment, and the AODV adapter defined here. Porting may change interfaces, signals, lifecycle semantics, or scheduling details; it therefore requires a new identity and compatibility record.

Deterministic probes cover the declared cases but not the full stochastic state space. Observation provenance and reporter identity remain assumptions at the simulator-model boundary. The zero-time reference run verifies composition and execution identity rather than network behavior, whereas the A2C13 runtime exercises the selected instrumentation and decision branches. Deployment on physical nodes would additionally require explicit mappings for clocks, concurrency, loss, radio behavior, and hardware resources.

The deterministic initialization records do not cover large radio networks, rapidly changing neighborhoods, competing discoveries or sustained mobility. These conditions may alter observation freshness, candidate turnover, pending forwarding state, event interleaving and the availability of a common basis. The authors’ catalogue and checkers can also share an interpretation error: agreement between them is not independent confirmation of the formal mapping. A checksum-verified record establishes byte identity, not algorithmic validity. Executable reproduction and independent analysis would strengthen confidence in the implementation mapping; separate native functional and comparative quantitative experiments are needed to support broader claims. No result from the planned third, fourth or fifth study is used as evidence for this manuscript.

## 14. Conclusions

The reported TA-BI implementation represents the declared observation-to-decision contract with typed evidence, versioned node and candidate state, separate score ownership, common-basis gating and native-first AODV advice. The authors’ deterministic tests qualify the stated implementation conditions. The retained final initialization probe supplies concrete decision and accounting counters at simulation time zero; it does not establish native-radio operation of the complete analytical chain.

The author-maintained catalogue records all 104 obligations as COMPLETE for the internal release. The code-free supplement preserves that verification record and supports integrity checking, but independent source rebuilding and third-party implementation verification remain unavailable. Network-level performance and robustness have not yet been validated. The current results therefore provide a test-based implementation baseline for the fixed reference profile. Broader functional behaviour, resource costs, scalability, mobility response and adversarial resilience require separate reproducible experiments. The planned third study addresses native functional validation; the fourth addresses quantitative comparison and resilience under declared conditions. A prospective fifth study addresses an original cross-study synthesis. No outcome of these future studies is assumed in the present conclusions.

## Figures and Tables

**Table 1:** Verified ownership in the completed reference implementation.

Executable Owner	Primary Responsibility	Explicit Exclusion
TaBiObservationModel, TaBiAodvAnalyzer	typed passive-event normalization and protocol mediation	no BI, Trust, RouteScore, or commit authority
NodeBehavioralAnalyzer	current-window BI and candidate Route Stability publication	no Trust, RouteBI, or RouteScore ownership
TrustManager, DeltaManager	node FR, LS, NR, Trust; historical updates only for FR and Trust	no destination-specific Trust or global smoothing
MainNodeBehavioralTable	BITReport validation, retained reports, NeighborBI	no transmitted reporter-Trust authority
BehavioralInformationTable	candidate versioning and RouteBI	no RouteScore or route commitment
RouteDecisionEngine	sole canonical RouteScore and typed readiness/audit	no native admissibility or route-table mutation
TaBiAodvDecisionAdapter	frozen native tie class and common-basis gate	provisional result only
TaBiAodvCommitController	native revalidation, hysteresis/dwell, atomic commit or fallback	no score-derived native metric penalty
TaBiLifecycleCoordinator	(t,p,s) ordering, immutable windows, atomic publication	no distributed/global-order claim
TaBiInstrumentationCollector/Ledger	separated counters and scalar export	no timer, decision mutator, gate, or routing authority

**Table 2:** Explicit crosswalk from the 18 Article 2 equations to the published Article 1 contract.

Article 2 Equation	Article 1 Authority	Relation
(1); (2)	Sections 3 and 4 and the evidence taxonomy	Authority and traceability.
(3); (4)	Equation (4) and Equation (55)	Typed evidence and state.
(5)	Equation (4), R-MISSING	Evidence eligibility.
(6); (7)	Equations (7)–(10), especially (9); Equations (19)–(20)	BI and NeighborBI mappings.
(8); (9)	Equations (40)–(41); Equations (21)–(23) and (29)	Forwarding evidence and candidate RouteBI mappings.
(10); (11)	Equations (46)–(51); Section 4.7 and Equations (52)–(54)	RouteScore and advice.
(12); (13)	Equation (66); Equation (70)	Frozen decision epoch and common component mask.
(14); (15)	Equations (71)–(73); Equations (76)–(79)	Complete common-basis gate and native-first ordering.
(16); (17); (18)	Equation (56), Algorithm 2, Proposition 7; Equation (57); evidence taxonomy	Outcome, event order and trace.

**Table 3:** Core formal-construct to verified executable-responsibility mapping.

Formal Construct	Executable Owner	Publication State	Primary Consumer
Packet-loss and delay evidence	NodeBehavioral Analyzer	observation/window records	BI construction
BI	NodeBehavioral Analyzer	NodeState.bi	RouteBI construction
Reported behavioral evidence	MainNodeBehavioral Table	reporter-aware records	NeighborBI aggregation
NeighborBI	MainNodeBehavioral Table	NodeState.neighborBi	RouteBI construction
FR, Link Stability, NR	TrustManager	node evidence records	raw Trust aggregation
Trust	TrustManager	NodeState.trust	RDE
Route Stability	NodeBehavioral Analyzer	CandidateState. routeStability	RouteBI construction
RouteBI	BehavioralInformation Table	CandidateState. routeBi	Route Decision Engine
RouteScore	Route Decision Engine	typed RouteAdvice	AODV adapter
Common basis and Γ	Adapter and epoch ledger	decision-epoch record	adapter ordering
Native tie class	Decision adapter	frozen candidate set	adapter ordering
Final revalidation	Commit controller	decision/fallback record	route commitment

**Table 4:** Traceability and release evidence for the internally controlled full A2 package.

Evidence Object	Coverage	Result
A2-REFSPEC-03_requirements.csv	normative catalogue plus final code/evidence/status columns	104/104 COMPLETE
A1_A2_CODE_TRACEABILITY.csv	Article 1 construct to concrete source mapping	104/104 MAPPED
A2_GLOBAL_CLOSURE_REGISTER.csv	controlled final disposition across A2C01–A2C13	104/104 COMPLETE
A2Cxx_TEST_MATRIX.csv and runtime registers	deterministic work-package checks and assertions	PASS
Terminal build/runtime evidence	clean release build, linkage, parent regression, reachability, raw outputs	PASS
IDE evidence	inet4.5 selected; legacy tabinet unselected; debug build	0 errors, 0 warnings
Package manifests	source, release inputs, file list, hashes, CRC and re-extraction	PASS

**Table 5:** Canonical parameter groups and exact released values.

Group	Released Values
BI	$w_{loss}=0.5$, $w_{delay}=0.5$, $D_{ref}=0.1$ s, $\kappa_{D}=1$, full-confidence counts 1, observation window 1 s
Historical update	coefficient bounds [0,1], $\delta_{FR}=0.25$, $\delta_{Trust}=0.5$
Trust	$\left(w_{FR},w_{LS},w_{NR}\right)=\left(0.5,0.3,0.2\right)$; age, confidence, loss-scale, and report-decay values fixed in the CSV contract
RouteBI	$\left(w_{BI},w_{NeighborBI},w_{RS}\right)=\left(0.4,0.3,0.3\right)$; node-evidence age 10 s; Route Stability age 2 s
RouteScore	$\left(w_{RouteBI},w_{Trust}\right)=\left(0.6,0.4\right)$; RouteBI age 2 s; Trust age 10 s
Decision/commit	minimum shared confidence 0; at most 64 candidates; epoch 1 s; hysteresis 0; dwell 0 s
Exclusions	TUNE-11, 12A, 13, 14A/14C, 15A, 16A, 17A plus controlled drop, synthetic traffic, and validation fixtures: all false
Instrumentation	instrumentationEnabled = true; collector/ledger remain observe-only

**Table 6:** Exact retained observations from the final initialization probe.

Recorded Quantity	Value	Meaning
Epochs opened/closed	1/1	controlled bounded epoch
Candidate admissions accepted/rejected	2/0	fixture candidate metadata
Tie-set size	2	one recorded tie-set observation
Gate open/closed	1/0	exercised path in this final probe
Provisional selections/commit invocations	1/1	controlled decision path
Winner revalidations valid/invalid	1/0	one provisional-winner check
Revalidated candidate total	2	separate candidate-total counter
Final commits/retentions/native fallbacks	1/0/0	recorded terminal outcome
BITReport lifecycle counters	1 each	six separately exercised accounting calls
Native/TA-BI logical messages	3/1	declared ledger stimuli
Native/TA-BI declared bytes	144/80	accounting inputs, not on-air measurements

## Data Availability

The original contributions presented in this study are included in the article/Supplementary Materials. Further inquiries can be directed to the corresponding author.

## Supplementary Materials

The following supporting information can be downloaded at https://www.mdpi.com/article/10.3390/s26206504/s1, Supplementary File S1: Publication evidence and traceability records for the TA-BI reference implementation in OMNeT++/INET, including verification and status reports, requirement and claim traceability, the Article 1–Article 2 equation crosswalk, the canonical decision contract, runtime outputs, terminal and IDE evidence, and SHA-256 integrity manifests.

## References

[1] Jabłoński W., Krenc K., Kawalec A. (2026). TA-BI: A Formal Observation-to-Decision Architecture for Trust-Aware Routing Support in Wireless and Mobile Wireless Sensor Networks. Sensors.

[2] Zhan G., Shi W., Deng J. (2012). Design and Implementation of TARF: A Trust-Aware Routing Framework for WSNs. IEEE Trans. Dependable Secur. Comput..

[3] Li X., Zhou F., Du J. (2013). LDTS: A Lightweight and Dependable Trust System for Clustered Wireless Sensor Networks. IEEE Trans. Inf. Forensics Secur..

[4] Varga A., Hornig R. An Overview of the OMNeT++ Simulation Environment. Proceedings of the 1st International Conference on Simulation Tools and Techniques for Communications, Networks and Systems & Workshops.

[5] INET Framework (2024). INET-4.5.4 Released. https://inet.omnetpp.org/2024-10-29-INET-4.5.4-released.html.

[6] INET Framework (2026). INET Framework: Open-Source OMNeT++ Model Suite for Wired, Wireless and Mobile Networks. https://inet.omnetpp.org/.

[7] Marti S., Giuli T.J., Lai K., Baker M. Mitigating Routing Misbehavior in Mobile Ad Hoc Networks. Proceedings of the 6th Annual International Conference on Mobile Computing and Networking.

[8] Michiardi P., Molva R., Jerman-Blažič B., Klobučar T. (2002). CORE: A Collaborative Reputation Mechanism to Enforce Node Cooperation in Mobile Ad Hoc Networks. Advanced Communications and Multimedia Security.

[9] Buchegger S., Le Boudec J.Y. Performance Analysis of the CONFIDANT Protocol. Proceedings of the 3rd ACM International Symposium on Mobile Ad Hoc Networking & Computing.

[10] Li X., Lyu M.R., Liu J. (2004). A Trust Model Based Routing Protocol for Secure Ad Hoc Networks. Proceedings of the 2004 IEEE Aerospace Conference.

[11] Li X., Jia Z., Zhang P., Zhang R., Wang H. (2010). Trust-Based On-Demand Multipath Routing in Mobile Ad Hoc Networks. IET Inf. Secur..

[12] Dhananjayan G., Subbiah J. (2016). T2AR: Trust-Aware Ad-Hoc Routing Protocol for MANET. SpringerPlus.

[13] Alzahrani A., Thomas N. (2024). Analysing the Performance of a Trust-Based AODV in the Presence of a Flooding Attack. Appl. Sci..

[14] Ye J., Jiang W. (2024). Routing Protocol for Underwater Wireless Sensor Networks Based on a Trust Model and Void-Avoided Algorithm. Sensors.

[15] Wang B., Ben K. (2024). GTR: GAN-Based Trusted Routing Algorithm for Underwater Wireless Sensor Networks. Sensors.

[16] Nagamani V., Annapurna K. (2025). Adaptive Clustering and Trust Aware Routing (ACTAR) for Wireless Sensor Networks in IoT Environments. Int. J. Commun. Syst..

[17] Jadidoleslamy H. (2025). A Distributed Fuzzy Trust-Aware Multipath Routing Protocol in Wireless Sensor Networks. Int. J. Commun. Syst..

[18] Banerjee B., Neogy S. (2026). Trust and Localization Adaptive Optimized Routing (TLAOR) in MANET. Peer-to-Peer Netw. Appl..

[19] Srinivasan V.P., Balachander K., Saravanan K. (2026). Intelligent Trust Assessment for Efficient Packet Transmission in MANETs Using Fuzzy Logic. Discov. Appl. Sci..

[20] Rady A., El-Rabaie E.S.M., Shokair M., Abdel-Salam N. (2021). Comprehensive Survey of Routing Protocols for Mobile Wireless Sensor Networks. Int. J. Commun. Syst..

[21] Burgos U., Amozarrain U., Gómez-Calzado C., Lafuente A. (2017). Routing in Mobile Wireless Sensor Networks: A Leader-Based Approach. Sensors.

[22] Faheem M., Gungor V.C. (2017). Capacity and Spectrum-Aware Communication Framework for Wireless Sensor Network-Based Smart Grid Applications. Comput. Stand. Interfaces.

[23] Perkins C.E., Belding-Royer E.M., Das S.R. (2003). Ad Hoc On-Demand Distance Vector (AODV) Routing.

[24] Bhargavan K., Obradovic D., Gunter C.A. (2002). Formal Verification of Standards for Distance Vector Routing Protocols. J. ACM.

[25] van Glabbeek R.J., Höfner P., Portmann M., Tan W.L. (2016). Modelling and Verifying the AODV Routing Protocol. Distrib. Comput..

[26] Bourke T., van Glabbeek R.J., Höfner P. (2014). A Mechanized Proof of Loop Freedom of the (Untimed) AODV Routing Protocol. Proceedings of the Automated Technology for Verification and Analysis.

[27] Kurkowski S., Camp T., Colagrosso M. (2005). MANET Simulation Studies: The Incredibles. ACM SIGMOBILE Mob. Comput. Commun. Rev..

[28] Huang Y., Cetinkaya D. (2026). Advancing Reproducibility and Replicability in Simulation: Challenges and Opportunities. Simulation.

[29] Virdis A., Stea G., Nardini G. (2014). SimuLTE: A Modular System-Level Simulator for LTE/LTE-A Networks Based on OMNeT++. Proceedings of the 4th International Conference on Simulation and Modeling Methodologies, Technologies and Applications.

[30] Nardini G., Sabella D., Stea G., Thakkar P., Virdis A. (2020). Simu5G: An OMNeT++ Library for End-to-End Performance Evaluation of 5G Networks. IEEE Access.

[31] OMNeT++ Community (2025). OMNeT++ 6.3.0 Available. https://omnetpp.org/software/2025/11/12/omnet-630-released.html.

[32] INET Framework (2026). Download and OMNeT++ Compatibility Information. https://inet.omnetpp.org/Download.html.

